# The role of astrocytes in neuropathic pain

**DOI:** 10.3389/fnmol.2022.1007889

**Published:** 2022-09-20

**Authors:** Tong Cheng, Zhongling Xu, Xiaqing Ma

**Affiliations:** Department of Anesthesiology, Affiliated Hospital of Nantong University, Medical School of Nantong University, Nantong, China

**Keywords:** astrocytes, neuropathic pain, signal transduction, cytokine, chemokine

## Abstract

Neuropathic pain, whose symptoms are characterized by spontaneous and irritation-induced painful sensations, is a condition that poses a global burden. Numerous neurotransmitters and other chemicals play a role in the emergence and maintenance of neuropathic pain, which is strongly correlated with common clinical challenges, such as chronic pain and depression. However, the mechanism underlying its occurrence and development has not yet been fully elucidated, thus rendering the use of traditional painkillers, such as non-steroidal anti-inflammatory medications and opioids, relatively ineffective in its treatment. Astrocytes, which are abundant and occupy the largest volume in the central nervous system, contribute to physiological and pathological situations. In recent years, an increasing number of researchers have claimed that astrocytes contribute indispensably to the occurrence and progression of neuropathic pain. The activation of reactive astrocytes involves a variety of signal transduction mechanisms and molecules. Signal molecules in cells, including intracellular kinases, channels, receptors, and transcription factors, tend to play a role in regulating post-injury pain once they exhibit pathological changes. In addition, astrocytes regulate neuropathic pain by releasing a series of mediators of different molecular weights, actively participating in the regulation of neurons and synapses, which are associated with the onset and general maintenance of neuropathic pain. This review summarizes the progress made in elucidating the mechanism underlying the involvement of astrocytes in neuropathic pain regulation.

## Introduction

Astrocytes or astroglia originate from radial glia cells that have been transformed from neuroepithelial precursor cells in the embryonic tube (Kriegstein and Noctor, 2004; Miller and Gauthier, 2007). As the most abundant and versatile glial cell type, accounting for approximately 20–40% of all glial cells in the central nervous system (CNS) (Colombo and Farina, 2016), astrocytes perform a broad range of different structural functions, including water and ion homeostasis, metabolism specialization, and brain oxidation, thus playing key roles in various levels of CNS function, such as growth, experience-dependent alignment, and maturation. In the absence of disease, astrocytes assist neurons metabolically and help sustain normal metabolic levels of extracellular K + ions, glutamate, and water. Astrocytes communicate with one another *via* hemichannels that join neighboring cells to construct intercellular gap junction communication pathways. As a result, astrocytes potentially transmit calcium waves and participate in the dissemination of various long-distance signaling molecules. Astrocytes also form a physical barrier around synapses, thus “shielding” them from glutamate overflow while simultaneously providing metabolic support. Furthermore, owing to their proximity to synapses, astrocytes modulate the local intervening ionic and chemical environment during synaptic formation and propagation. Thus, astrocytes play a pivotal role in governing synaptic formation, maturation, removal, and maintenance as well as supporting synaptic activity *via* a combination of diffusible and interaction factors. Loss-of-function disorders of astrocytes may constitute the basis of various forms of neurological dysfunction and pathology, including trauma, stroke, multiple sclerosis, and Alzheimer’s disease (Huynh et al., 1997; Weggen et al., 1998; Simpson et al., 2010).

Nociception is recognized as a negative sensorial feeling that is related to or described in terms of real or prospective tissue injury. While acute pain possesses an important protective function, chronic pain is often maladaptive, with no biological benefits. Chronic pain refers to a pain state that lasts for more than 1 month, and it can be derived from several different etiologies. An epidemiological study revealed that the prevalence of chronic pain in China was approximately 31.54%. Other studies have demonstrated that it affects up to 30% of adults worldwide and costs the United States economy over $600 billion per annum (Bouhassira et al., 2008). The long duration of hyperalgesia and ectopic pain has a negative impact on individuals’ mental state and enjoyment; therefore, it qualifies as one of the major predisposing factors leading to depression and even suicide. Neuropathic pain is a kind of chronic pain that is common and greatly affects patients’ lives, and it is defined by the International Association for the Study of Pain as “pain generated by a lesion or disease of the somatosensory nervous system” (Bouhassira, 2019). A considerable amount of research has been conducted on neuropathic pain in past decades, and it has generally been regarded a consequence of the complex mutual effect of mechanisms in the peripheral and central nervous systems. Importantly, consensus has increasingly corroborated the peculiar roles of reactive glial cells in the onset and persistence of neuropathic pain.

## Overview of neuropathic pain

Pain can be perceived as a self-protective mechanism of the body, warning the body of ongoing or impending tissue damage. For example, the pain sensation that occurs when a finger is scraped by a knife is nociceptive pain. If the tissue is damaged, the body will follow up with an inflammatory response, which promotes tissue repair while releasing various mediators that stimulate injury receptors and cause pain. This pain is known as inflammatory pain. In contrast, neuropathic pain is defined as pain produced by injury or disease affecting the somatosensory nervous system and is distinguished by the following clinical presentations: spontaneous pain, nociceptive hyperalgesia, and pain triggered by touch. Neuropathic pain generally affects people more and lasts longer than other types (Torrance et al., 2006) of pain.

Neuropathic pain is typically characterized by both positive and negative sensory symptoms. Positive sensory symptoms include spontaneous positive sensations, such as paresthesia, dysesthesia, paroxysmal pain, and persistent superficial pain, and stimuli-induced positive sensations involving hyperalgesia and allodynia. Mechanical hyperalgesia, typically characterized by a mildly painful, prick-like, irritating stimulation, potentially leads to a more painful sensation. Mechanical dynamic nociception and mechanical static nociception are two terms used to characterize painful conditions caused by slight moving touch and pressure, respectively. In addition, defects in different somatosensory characteristics, including, but not limited to, numbness, deadness, tactile hypoesthesia, reduced heat sensitivity, and lack of vibratory feeling, although habitually overlooked, constitute manifestations of the negative symptoms of neuropathic pain.

Several different mechanisms that are required for neuropathic pain to develop are not limited to the periphery alone, but even extend all the way to the CNS, including the spinal cord, brain, and downstream modulatory system. These mechanisms have preliminarily been explored (Cohen and Mao, 2014). Several maladaptive mechanisms underlying these symptoms not only comprise peripheral hypersensitivity of nociception and hyperexcitability of afferent neurons but also central hypersensitivity, including vicarious pain facilitation, de-suppression of pain sensation, and central restructuring procedures, in addition to sympathetic persistent pain.

To study the occurrence and development of pain, highlight novel pain signaling pathways, and solve emerging issues in the field of pain, such as the development of more effective analgesic drugs and avoidance of adverse effects induced by analgesic drugs, several rodent models for pain research have been established to imitate human pain circumstances. In the course of neuropathic pain research, surgical models are crucial in establishing nociceptive states, predominantly comprising chronic constriction injury (CCI) to the sciatic nerve (Bridges et al., 2001; Hogan, 2002), partial sciatic nerve ligation (Seltzer et al., 1990), spinal nerve ligation (Challa, 2015), spare nerve injury (SNI) (Erichsen and Blackburn-Munro, 2002; Rode et al., 2005; Bourquin et al., 2006), brachial plexus avulsion (Carvalho et al., 1997; Rodrigues-Filho et al., 2003, 2004), spinal nerve transection (SNT) (Li et al., 2002), and sciatic nerve transection models (Wall et al., 1979). Most of these models induced reactions comparable to those reported in causal pain, a persistent burning pain condition commonly exhibited in human distal limbs following damage to certain peripheral nerves. Under normal conditions, investigators apply different forms of pain to rodents in research projects to identify novel molecular substances and attain commendable achievements in the field of neuropathic pain (Challa, 2015).

## Overview of astrocytes

In the CNS, glial cells outnumber neurons by a factor of 10–50, predominantly including astrocytes, microglia, and oligodendrocytes (Moalem and Tracey, 2006). Astrocytes are regarded the most numerous cells in terms of number and volume, accounting for >40% of all glial cells (Aldskogius and Kozlova, 1998). As a class of neural cells originating from the ectoderm and neurepithelium, a single astrocyte is estimated to be capable of wrapping around 140,000 synapses and four to six neuron somata and contacting 300–600 neuron dendrites in rodents (Bushong et al., 2002; Oberheim et al., 2009; Gao and Ji, 2010b). With these physiological strengths, astrocytes sustain homeostasis and are partly responsible for defending the CNS (Verkhratsky and Nedergaard, 2018).

### Physiological functions of astrocytes

Utilizing multiple biologically based techniques and state-of-the-art *in vivo* non-traumatic approaches (e.g., positron emission tomography, magnetic resonance imaging scans, and magnetic resonance spectroscopy), astrocytes have been thoroughly studied, and their roles encompass a broad variety of dynamic physiological compositions and functionalized features: water and ion homeostasis, metabolic specialization, and brain oxidation (Volterra and Meldolesi, 2005; Haydon and Carmignoto, 2006; Romero-Sandoval et al., 2008; Fellin, 2009; Jiang et al., 2020).

**FIGURE 1:**
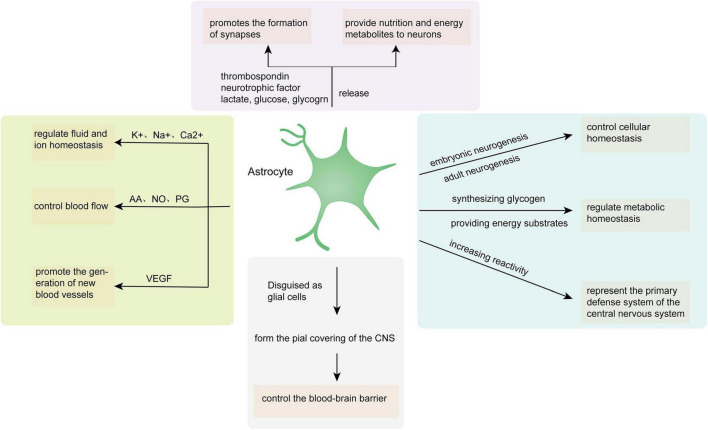
Astrocyte functions in the CNS. AA, arachidonic acid; NO, nitric oxide; PG, prostaglandin; VEGF, vascular endothelial growth factor.

Astrocytes play a role in neural tissue, closely combining with neural networks, and control the homeostatic balance between individual molecules in the CNS and individual tissues of the entire organ through the transportation of major ions and protons, clearance and decomposition of neurotransmitters, and release of neurotransmitter precursors and reactive oxygen scavengers. Astrocytes maintain neurotransmission as a consequence of providing neurotransmitter precursors for neurons and control cellular homeostasis through embryonic and adult neurogenesis. Moreover, regulating metabolic homeostasis by synthesizing glycogen and providing energy substrates for neurons is also a momentous routine of astrocytes. Disguised as glial cells, astrocytes constitute a cortical covering that envelops the CNS, manage the blood-brain barrier, and serve as chemoreceptors, thereby promoting dynamic homeostasis throughout the body. Traumatic brain injury, infection, and other diseases can increase astrocyte reactivity, thus transforming resting astrocytes into reactive astrocytes with abnormal characteristic behavior. Thus, astrocytes potentially represent the primary defense system of the CNS by increasing reactivity (Figure 1).

These routine structural, functional, and physiological strengths of astrocytes play crucial roles in various levels of CNS function, such as growth, experience-dependent alignment, and maturation (Verkhratsky and Nedergaard, 2018; Tsuda, 2019; Giovannoni and Quintana, 2020).

#### The role of astrocytes in synaptic transmission and synaptic plasticity

The special, close relationship between astrocyte membranes and synaptic structures, the high expression of neurotransmitters in astrocytes, and their regulation of synaptic transmission concertedly create a tripartite synaptic model (Araque et al., 1999; Halassa et al., 2007). Astrocytes potentially serve as a multifunctional key in controlling all aspects of synaptic connections: regulating the maturation of the synaptic network; maintaining the stability of the ionic dynamics of the synaptic cleft; monitoring and regulating the constant changes in neurotransmitters; and ultimately, participating in synaptic extinction (Nedergaard and Verkhratsky, 2012; Verkhratsky and Nedergaard, 2014). In addition, during synaptogenesis, astrocytes surprisingly possess a considerable degree of flexibility and mobility that helps stabilize new synapses. As the close relationship between astrocytes and synapses becomes better understood and more deeply studied, further possibilities for astrocytes in synaptic transmission and plasticity are unfolding (Barker and Ullian, 2010).

#### The role of astrocytes in memory

Astrocytes have been proven to be intimately involved in neuronal functions in memory formation (Haydon and Nedergaard, 2014; Moraga-Amaro et al., 2014). Suzuki et al. (2011) demonstrated that astrocyte glycogenolysis and lactate release contribute to the formation of long-term memory and maintenance of long-term enhancement of synaptic strength induced *in vivo*. In addition, we have learned that astrocyte-derived lactate produced by glycogenolysis is involved in regulating the changes in substances required for long-term memory formation, including, but not limited to, the expression of target genes and phosphorylation of the transcription factor CREB, among others, thus contributing significantly to long-term memory formation in rats (Suzuki et al., 2011).

### Astrocytes in pathology

During the development of neurological diseases, astrocytes often exhibit different pathological manifestations successively or simultaneously, such as, dysfunctional astrocyte atrophy, pathological remodeling of astrocytes, and reactive astrocyte hyperplasia (Pekny et al., 2016). Disorders or loss of function may form the basis of various forms of neurological dysfunction and pathology, including, but not limited to, trauma, stroke, multiple sclerosis, and Alzheimer’s disease, among others (Weggen et al., 1998; Simpson et al., 2010).

In addition, they have been linked to a worse prognosis of CNS injury, such as excitatory toxic neurodegeneration due to inadequate glutamate intake (Bush et al., 1999; Swanson et al., 2004) or a higher risk of invasion or injury resulting from astrocyte barrier dysfunction (Bush et al., 1999; Faulkner et al., 2004; Drögemüller et al., 2008; Herrmann et al., 2008; Voskuhl et al., 2009). Extensive molecular and cellular biological evidence has currently demonstrated that the harmful effects of reactive astrogliosis and glial scarring are typically manifested by the inhibition of axon regeneration (Silver and Miller, 2004). Furthermore, astrocytes have appeared in a variety of domestic and international studies on different disease pathological states (Swanson et al., 2004; Brambilla et al., 2005; Jansen et al., 2005; Tian et al., 2005; Hamby et al., 2006; Farina et al., 2007; Brambilla et al., 2009). Some researchers have that found seizures in epilepsy may be directly related to the dysfunction and denaturation of astrocytes triggered by abnormalities in cytotoxic T lymphocytic autoimmunity (Whitney and McNamara, 2000; Bauer et al., 2007). Other autoimmune inflammatory states involving the CNS have also been reasonably speculated to be associated with astrocytes, such as systemic lupus erythematosus; however, they have not been explicitly studied (Tomita et al., 2004). Furthermore, astrocytes have now been firmly considered to make a significant contribution to the pathophysiology of hepatic encephalopathy (Norenberg et al., 2005).

## Activation of astrocytes in neuropathic pain

Astrocyte activation has been identified within a few layers of the CNS, such as the spinal cord and supraspinal center (Gao and Ji, 2010b). In fact, current research on the relationship between astrocytes and pain has predominantly focused on the spinal cord level. In pain research, astrocyte activation is commonly understood as the increased expression of the glial fibrillary acidic protein (GFAP) or astrogliosis.

**FIGURE 2:**
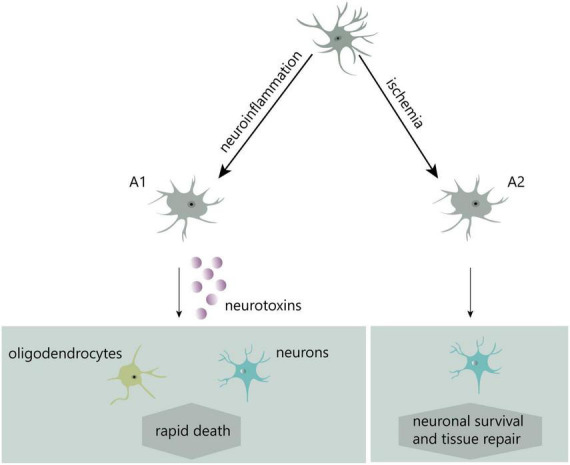
Two different types of reactive astrocytes.

In recent years, two distinct kinds of reactive astrocytes, A1-reactive and A2-reactive astrocytes, have been identified (Liddelow et al., 2017). Induced by neuroinflammation, A1 astrocytes potentially secrete neurotoxins leading to rapid death of neurons and other types of glial cells. Conversely, ischemia-induced A2 astrocytes contribute to more effective tissue healing and neuronal preservation (Liddelow et al., 2017; Figure 2). A1-reactive astrocytes rather than A2 are mostly believed to be involved in the development of neuropathic pain, and much of the research in this area has focused on them.

Astrocytes from the CNS, particularly the spinal cord, have been shown to undergo morphophysiological and behavioral alterations, such as translation and transcription, following peripheral nerve injury, inflammation, or tumor invasion. Certain activation states, such as an increase in intracellular Ca2+ flux and phosphorylation of signaling molecules, are considered to be completed within minutes. Activation states that take tens of minutes (translation regulation) or hours (transcriptional regulation) to follow also exist. Longer activation states, such as astrogliosis, may require longer periods of time, such as tens of hours or even days to appear (Gao and Ji, 2010b).

Astrocytes are generally believed to regulate pain mainly through the following three activation states: (1) changes in glial signaling pathways, such as changes in the phosphorylation level of mitogen-activated protein kinase (MAPK) and expression of transcription factors; (2) changes in the expression of receptors and channel proteins, such as the upregulation of inflammatory factor receptors and gap junction proteins and downregulation of glutamate transporters; and (3) continuous production and excretion of various glia-derived factors, such as cellular factors, chemokines, and proteinases (Gao and Ji, 2010b; Ji et al., 2013).

Hofstetter et al. (2005) claimed that the transplantation of neural stem cells that can transform into astrocytes into damaged spinal cords resulted in an abnormal, pain-like hypersensitivity response in the front paw. Furthermore, the inhibition of astrocyte differentiation of transplanted cells by transducing neural stem cells with neurogenin-2 before transplanting potentially prevents graft-induced abnormal pain. Inhibitors of glial cells and astrocytes, in particular, fluoroacetate and its metabolite fluorocitrate (FC), only require considerably minute quantities to specifically interfere with astrocyte physiological metabolism *via* obstructing a glial cell enzyme in the astrocytic Krebs cycle. Several mouse models of pain that had undergone intrathecal injection of FC or fluoroacetate demonstrated relief from pain sensation (Milligan et al., 2003; Obata et al., 2006; Clark et al., 2007; Okada-Ogawa et al., 2009). In addition, astrocyte engagement in the ventrolateral periaqueductal gray (vlPAG) was found to be closely associated with pain response. Liu et al. (2022) injected FC into the vlPAG of diabetic neuropathic pain (DNP) rats and found the mechanical withdrawal threshold of DNP rats to decrease significantly, suggesting that the pain-relieving action of FC in DNP rats was related to the inhibition of astrocyte activation in the vlPAG. FC’s pain-relieving action has been frequently validated in a wide range of different research topics on neuropathic pain, revealing a close relationship between astrocyte activation and neuropathic pain status.

## Key signaling mechanisms and molecules regulating reactive astrocytes

**FIGURE 3:**
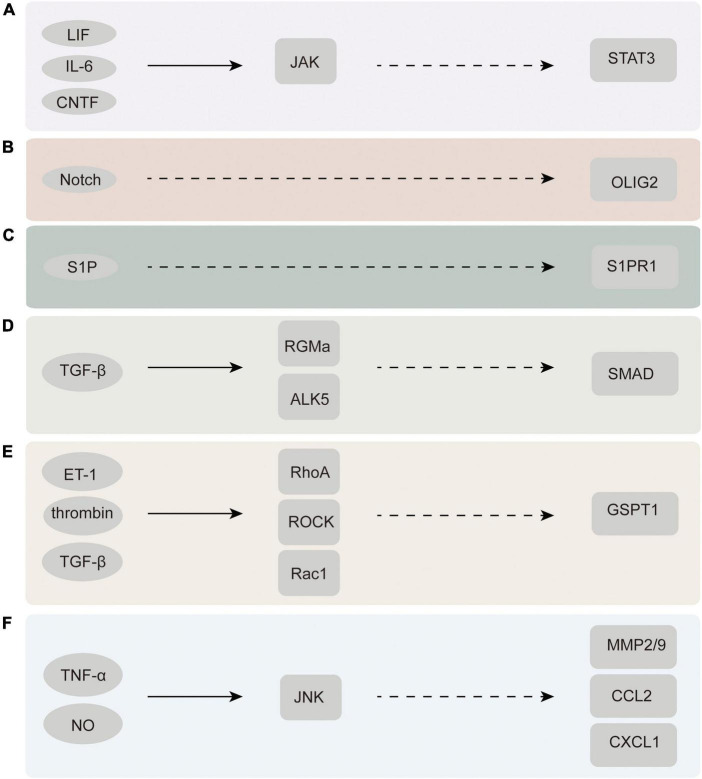
Chemicals and molecules that activate astrocytes and their upstream and downstream signaling pathways. **(A)** JAK-STAT3 signaling pathway. **(B)** Notch-Olig2 signaling pathway. **(C)** S1PR1 signaling pathway. **(D)** TGF-β-SMAD signaling pathway. **(E)** Rac1-GSPT1 signaling pathway. **(F)** JNK signaling pathway. LIF, leukemia inhibitory factor; CNTF, ciliary neurotrophic factor; ET-1, endothelin-1; ROCK, rho associated kinase; GSPT1, G1 to S phase transition 1; JNK, C-Jun N-terminal kinase; MMP2/9, matrix metalloproteinase-2/9.

Multiple signal transduction pathways are engaged in the process of converting resting astrocytes into reactive astrocytes (Karimi-Abdolrezaee and Billakanti, 2012). Many diverse cell types, such as neurons, glial cells, and inflammatory cells, release chemical signals that stimulate nascent astrocytes (Pekny et al., 2016). Chemical signals engaged in the regulation of astrocyte activation comprise proinflammatory cytokines [interleukin-1β (IL-1β), tumor necrosis factor α (TNF-α), and interleukin-6 (IL-6)], and gene transcription factors [signal transduction and transcriptional activator 3 (STAT3)], among others (Koyama, 2014; Figure 3).

### Janus kinase-signal transduction and transcriptional activator 3 pathway

Tsuda demonstrated that astrocyte proliferation in neuropathic pain is regulated by the JAK-STAT3 signaling pathway. Moreover, the application of the janus kinase (JAK) inhibitors AG490 and JAK inhibitor I to the spine resulted in the inhibition of astrocyte proliferation during astrocyte restriction and STAT3 activation. Furthermore, the inhibition of astrocyte proliferation by JAK-STAT3 signaling inhibitors potentially leads to the recovery of haptic pain (Tsuda et al., 2011). Therefore, it is reasonable to conclude that the inhibition of JAK-STAT3 signaling leads to that of astrocyte proliferation in the dorsal horn.

Some experiments have demonstrated that the proliferative capacity of astrocytes intervened by AG490 or with STAT3 defects is reduced to a certain extent (Washburn and Neary, 2006; Sarafian et al., 2010). Liu et al. (2021) demonstrated that not only were the activation levels of JAK2 and STAT3 significantly reduced by miR-135-5p mimics intervention but also GFAP, IL-1β, and TNF-α expression, which was consistently decreased. Moreover, functional *in vitro* studies have also confirmed that miR-135-5p potentially inhibits the JAK2-STAT3 signaling pathway in bone cancer pain, and histological staining has proven that miR-135-5p can directly interrupt the activity process of JAK2 and its upstream and downstream molecules in astrocytes (Liu et al., 2021). These studies demonstrate that JAK-STAT3 signaling has a key contribution in the occurrence and progression of neuropathic pain *via* the modulation of astrocyte activation.

### Notch–oligodendrocyte transcription factor 2 pathway

Oligodendrocyte transcription factor 2 (Olig2) is an essential transcription factor required for the proliferation and derivation of developmental astrocytes. Olig2-labeled subsets of astrocytes have been detected in clusters and in greater numbers in certain fixed zones of the thalamus, midbrain, and spinal cord of rodents (Wang et al., 2021). By ablating Olig2, Chen found reactive astrocyte proliferation to be reduced, demonstrating that Olig2 plays a key role in reactive astrocyte proliferation (Chen et al., 2008). A BrdU labeling experiment suggested that Notch molecules may contribute to the numerical increase and functional specialization of reactive astrocytes by regulating the nuclear cytoplasmic translocation of Olig2 (Marumo et al., 2013). Mice with exclusive Notch 1 knockout in GFAP-positive reactive astrocytes also exhibited a significant reduction in proliferative reactive astrocytes (Shimada et al., 2011). Both mechanical and thermal hyperalgesia induced by CCI were effectively reduced by inhibiting the mRNA expression of Olig2, a marker of astrocyte activation (Bertozzi et al., 2017). The above findings suggest that the Olig2 signaling pathway is highly involved in astrocyte activation and the progression of neuropathic pain.

### S1P receptor subtype 1 pathway

Chen et al. (2019) found that sphingosine-1-phosphate (S1P), which is produced in the spinal dorsal horn of CCI and SNI models, actuates neuropathic pain through targeted activation of S1P receptor subtype 1 (S1PR1) present in astrocytes. CCI and SNI models treated with an astrocyte-specific S1PR1 knockout were not observed to experience neuropathic pain. Therefore, astrocytes have been identified as the major cellular substrates for S1PR1 activity. In addition, their study also found that the relief of neuropathic pain induced by S1PR1 inhibition is mediated by interleukin-10 (IL-10), a cellular factor capable of participating in neuroprotection and fighting against inflammatory infections, thus identifying the S1PR1 axis as a key link in the developmental process and signaling pathways of neuropathic pain (Chen et al., 2019). Therefore, S1PR1 is expected to be a novel therapeutic target for neuropathic pain.

### Transforming growth factor beta- Sma- and Mad-related protein pathway

Transforming growth factor betas (TGF-βs), as versatile growth factors, are involved in critical events in the body that regulate growth, illness, and wound healing. TGF-β1 has been extensively known to be a cytokine implicated in injury, especially in the activation of astrocytes and scar production (Diniz et al., 2019). TGFβ is immediately triggered following CNS damage and stimulates the Sma- and Mad-related protein (SMAD) family of transcription factors in astrocytes (Koyama, 2014). Ralay Ranaivo et al. (2010) successfully activated the SMAD pathway downstream of TGFβR using albumin in primary astrocyte cultures. Other studies have found that the knockdown of repulsive guidance molecule a (RGMa) eliminates TGFβ1-induced astrocyte proliferation and activation and proved that RGMa potentially promotes TGFβ1/SMAD2/3 signal transduction by binding to activin receptor-like kinase 5 and SMAD2/3 simultaneously, constituting a composite (Zhang et al., 2018). The above findings suggest that the TGF-SMAD signaling pathway is expected to become an important target for astrocyte activation and neuropathic pain treatment.

### Rac1–G1 to S phase transition 1 pathway

As a member of the Ras homolog gene family, the small G protein RhoA is widely expressed in astrocytes and participates in cytoskeletal regulation together with its downstream effector Rho-dependent kinase (ROCK) by regulating myosin and actin (Tönges et al., 2011; Maldonado et al., 2017; Wen et al., 2019; Lu et al., 2021). The expression of RhoA and Rac1 in the glial scar area is significantly increased after injury (Erschbamer et al., 2005). Several experimental results reveal that the activation of RhoA/ROCK and its upstream and downstream molecules facilitates the occurrence and sustainment of neuropathic pain (Tatsumi et al., 2005; Hang et al., 2013). The inhibition of ROCK leads to rapid and reversible astral alignment of cultured astrocytes and increases their migratory activity. In one experiment, Rac1-KD or Rac1-KO astrocytes displayed significantly delayed cell cycle progression and decreased cell migration as well as the diminished expression of G1 to S phase transition 1 (GSPT1). In addition, the decreased expression and response of GSPT1 to lipopolysaccharide (LPS) treatment were observed. This suggests that GSPT1 is a downstream target of Rac1 (Ishii et al., 2017). Rac1-GSPT1, as a novel signaling axis in astrocytes, is an important factor in astrocyte activation after CNS injury. However, whether GSPT1 directly leads to neuropathic pain still warrants further research.

## Intracellular kinases, channels, transporters, and receptors involved in neuropathic pain regulation in astrocytes

Many signaling molecules exist in astrocytes, including intracellular kinases, channels, receptors, and transcription factors, exhibiting pathological changes and playing a role in pain regulation after injury.

### Intracellular kinases

The MAPK family, which is composed of P38, extracellular signal-regulated kinase (ERK), and C-Jun N-terminal kinase (JNK), plays a crucial role in pain regulation (Gao and Ji, 2010b). Different members exhibit specific expressions in various pain states (Ji et al., 2009); nonetheless, all of them have been proven effective in alleviating neuropathic pain in various pain models (Zhuang et al., 2005; Gao et al., 2009; Wang et al., 2012).

Phosphorylated ERK has been observed in spinal astrocytes during the late stage of pain induced by complete Freund’s adjuvant (Weyerbacher et al., 2010). In an experiment, Yu used the mitogen-activated ERK kinase inhibitor U0126 to inhibit astrocyte activation and moderate the release of proinflammatory cytokines (IL-1β and IL-6) by activated astrocytes. The ERK1/2 pathway was found to potentially mediate astrocyte activity through the PPARγ pathway in CCI rats (Zhong et al., 2019).

Choi et al. (2019) demonstrated that the neurosteroid-metabolizing enzyme cytochrome P450c17 stimulates astrocytes through the P38 phosphorylation pathway, eventually contributing to the occurrence of CCI-induced mechanical nociception in mice.

Janus kinase has been shown to engage in the regulation and sustainment of nociception by astrocytes after nerve damage (Cao et al., 2015). P-JNK inhibitors relieve neuropathic pain in mice by inhibiting JNK signaling in spinal cord astrocytes (Li et al., 2020). Tetramethylpyrazine selectively inhibits JNK activity, inhibits astrocyte activation, and reduces matrix metalloproteinase-2/9 (MMP-2/9) expression, thus alleviating the maintenance of neuropathic pain (Jiang et al., 2017). This suggests that JNK-MMP-2/9 is a potentially promising new focus for neuropathic pain relief.

### Receptors

To date, relatively few ion channels and receptors that are involved in the activation of astrocytes, thus ultimately leading to the development of neuropathic pain, have been identified (Table 1).

**TABLE 1:** Expression of ion channels and receptors in astrocytes.

Classifications	Name	Ligands	Mediators regulated *via* activation	Changes in expression in neuropathic pain	References
Ion channels	Connexin-43	Cx43 siRNA, Cx43 mimetic peptide, Ca_V_3.2, CaMKII	CXCL12, LPS, IL-1β, IL-6, c-fos, ATP	Up-regulated	Gao and Ji, 2010b; Li et al., 2021; Zhu et al., 2022
	Connexin-30	–	IL-1β, IL-6	Up-regulated	Gao and Ji, 2010b
	AQP4	Anti-AQP4 recombinant autoantibodies (rAQP4 IgG), TGN-020, TNF-α	ATP, glutamate transporter 1, c-fos, ERK	Up-regulated	Wang et al., 2020; Ishikura et al., 2021; Guo et al., 2022
Receptors	IL-18 receptor	IL-18	Nuclear factor kappaB	Up-regulated	Miyoshi et al., 2008
	TLR2	Triacylated lipoproteins, peptidoglycan glycolipids, heat shock protein-60, heat shock protein-70	MCP-1, reactive oxygen species, CXCL8, NO, IL-6	Up-regulated	Bowman et al., 2003; Kawai and Akira, 2009; Milligan and Watkins, 2009
	TLR4	LPS, hyaluronate, envprost, heparin, taxol, HMGB1	MCP-1, TNF-α, IL-6, NO, IL-1α, IL-1β,	Up-regulated	Scholz and Woolf, 2007; Nicotra et al., 2012; Thakur et al., 2015
	Trkb.T1	BDNF, ligands of the G-protein-coupled receptor (GPCR) family of transmembrane receptors	Rho GDP dissociation inhibitor, Ca2 +	Up-regulated	Matyas et al., 2017; Cao et al., 2020

Toll-like receptor 4 (TLR-4) is widely present throughout astrocytes. Overexpression of the high mobility group box-1 (HMGB1) protein potentially downregulates the paw withdrawal mechanical threshold and paw withdrawal thermal latency in CCI rodents by enhancing the activation of astrocytes, the TLR4/NF-κB axis, and its upstream and downstream molecules, resulting in the enhancement of neuropathic pain (Zhao et al., 2020). A subject of experimental observations in mice treated with toll-like receptor 2 (TLR2) knockout demonstrated that TLR2 expression is essential for neuropathic pain development. Neurodamage-induced astrocyte activation was also reduced in TLR2-knockout mice. The expression of TLRs has been found to enable astrocytes to release inflammatory mediators closely related to neuropathic pain, including, but not limited to, IL-6, monocyte chemoattractant protein-1 (MCP-1), and nitric oxide (NO) (Thakur et al., 2017). Thus, both TLR2 and TLR4 facilitate the progression and sustainment of pain caused by nerve damage (Kim et al., 2007).

A shortened isomeric form of tyrosine receptor kinase B (TrkB) has recently been found to be implicated in the pathological process of neuropathic pain (Renn et al., 2009). The TrkB.T1 isomeric form is generated through alternative splicing of TrkB. As the only isoform expressed in astrocytes (Rose et al., 2003; Dorsey et al., 2006; Ohira et al., 2007; Holt et al., 2019), Trkb.T1 has exhibited an increased expression in various experimental models of pain (Renn et al., 2009; Wu et al., 2013; Matyas et al., 2017) and attracted increasing attention. Antiretroviral treatment causes abnormal mechanical pain in mice through triggering the activity of brain-derived neurotrophic factor (BDNF) and consequently resulting in polyneuronal hyperactivity (Renn et al., 2011). Trkb.t1-specific knockout mice (Dorsey et al., 2006) were found to be relieved of abnormal pain sensation and heat hyperalgesia in the neuropathic pain model system (Renn et al., 2009). After RNA sequencing work on astrocytes extracted from TrkB.T1 KO mice, the researchers detected a downregulation in the ability of their astrocytes to divide, differentiate, and migrate (Cao et al., 2020). These results suggest that the imbalance in the physiological state of astrocytes caused by the involvement of TrkB.T1 promotes neuropathic pain development.

Considering the prevalence of neuropathic pain and incurable suffering associated with it, the study of ion channels and receptors associated with astrocytes is a potentially new opportunity to bring hope to affected people worldwide. AV411 (ibudilast), an antagonist of TLR4, is currently undergoing phase II clinical trials and has previously been shown to exert satisfactory efficacy in various models of neuropathic pain by inhibiting cytokines produced by glial cells as well as promoting the production of the anti-inflammatory cytokine IL-10 and neurotrophic factors (Connolly and O’Neill, 2012). This presents new directions and possibilities to research on targeted therapy for neuropathic pain. The TLR family and Trkb.T1 deserve further investigation and deeper exploration as key components of astrocytes that are closely linked to neuropathic pain.

### Gap junctions

Astrocytes form a network of gap junctions through Ca2+ signals in the form of calcium oscillations (Blomstrand et al., 1999; Haydon, 2001). The main structural components of gap junctions are connexins (CX), such as Cx26, Cx29, Cx30, Cx32, and Cx36, among others. Cx30 and Cx43 are specifically expressed in astrocytes (Giaume and McCarthy, 1996; Nagy et al., 2004). Astrocytic connexins not only regulate gap junction function but also act as semi-channels to transfer and release chemicals such as ATP from inside to outside the cell (Xing et al., 2019), thus regulating synaptic transmission and further leading to pain by directly interacting with injurious neurons (D’Hondt et al., 2014). Cx43 has been shown to effectively regulate the production and release of the chemokine CXCL12 in bone marrow stromal cells (Schajnovitz et al., 2011). Transfection of Cx43 can inhibit LPS-triggered hyperexcitability and upregulation of proinflammatory cellular factors and chemokines (Tsuchida et al., 2013). In addition, intrathecal injection of HIV1 GP120 is followed by a massive proliferation of IL-1β and IL-6 in the cerebrospinal fluid. Carboxy ketone (CBX), a non-specific gap junction antagonist, can block this in a timely and effective manner (Spataro et al., 2004). These behaviors are directly related to neuropathic pain.

## Astrocyte-derived inflammatory mediators involved in neuropathic pain regulation

Astrocytes regulate neuropathic pain by releasing a series of mediators of different molecular weights. Macromolecular mediators are represented by cellular factors (e.g., TNF-α and IL-1β), chemokines (e.g., CCL2 and CXCL1), growth factors (e.g., BDNF and bFGF), and proteases (e.g., MMP-2 and tPA), among others. Small molecular media predominantly comprise glutamic acid and ATP, among others. These mediators actively participate in the regulation of neurons and synapses and are inextricably linked to the onset and maintenance of neuropathic pain.

### Proinflammatory cytokines

IL-1β is a well-known proinflammatory cytokine. IL-1β upregulation has been observed in spinal cord astrocytes following peripheral nerve lesions. Increasing evidence suggests that IL-1β is involved in pain sensitivity (Cao and Zhang, 2008; Kiguchi et al., 2012). Intrathecal administration of IL-1β receptor antagonists alleviates inflammatory and neuropathic pain. Neuropathic pain was also significantly reduced in mice with IL-1β type I receptor knockout or those deliberately treated with an IL-1β antagonist. In contrast, IL-1β injected into the spinal canal induces pain hypersensitivity (Gao and Ji, 2010b; Ji et al., 2013). Further experiments have also shown that IL-1β is directly sensitive to thermally and chemically sensitive cationic channels in the transient receptor potential cation channel subfamily V member 1 (Kiguchi et al., 2012).

### Chemokines

Chemokines, a specific group of cell factors possessing over 50 family components, are widely believed to exist in the blood and immune system and have recently been found to be widespread in the CNS and particularly active in astrocytes (Kiguchi et al., 2012). Chemokines predominantly perform their biological functions by binding to their G-protein-coupled receptors. Current studies have demonstrated that chemokines and corresponding receptors are usually present in different cell types to participate in their interaction and regulation (Gao and Ji, 2010a). CCL2, also called MCP-1, is mainly derived from astrocytes. A remarkable increase in JNK-dependent CCL2 in spinal astrocytes induced by peripheral nerve injury has also been observed, and it has been found to drive neuropathic pain with direct modulation of spinal neurons. CCL2 can rapidly increase the inward currents of spinal dorsal horn neurons induced by AMPA and NMDA. This indicates increased synaptic glutamate transmission, which is strongly associated with increased central sensitization and markedly diminished nociceptive sensation (Gao and Ji, 2010b; Nakagawa and Kaneko, 2010). Furthermore, CCL2 potentially stimulates the division and differentiation of spinal microglia to further support pain development (Nakagawa and Kaneko, 2010).

CCL3 has also been found to cause neuropathic pain through IL-1β upregulation (Kiguchi et al., 2012). CXCL1, an essential cytokine, potentially acts as a mediator, lubricating the communication between neurons and astrocytes (Kiguchi et al., 2012). In spinal nerve ligation models, CXCL1 and its receptor CXCR2 have been observed to undergo upregulation in spinal astrocytes and neurons (Zhang et al., 2013). Intrathecal injection of anti-CXCl1 antibodies has been found to successfully alleviate pain hypersensitivity induced by spinal nerve ligation, and CXCR2 antagonists can definitively terminate CXCl1-induced thermal pain allergy. This implies that CXCL1, CXCR2, and CCL3 are all involved in driving neuropathic pain.

### Proteases

Proteases expressed in astrocytes, such as matrix metalloproteinases (MMPs) and tissue-type plasminogen activators (tPAs), have been found to play a key role in the development of neuropathic pain driven by astrocytes (Gao and Ji, 2010b). Different MMPs have been implicated in the progression of neuropathic pain at different periods of time after nerve injury. MMP-9 induces the early development of neuropathic pain by mediating the early cleavage of IL-1β and activation of microglia, while MMP-2 is engaged in the sustainment of neuropathic pain in late stages through modulating the cleavage of IL-1β and activation of astrocytes (Kawasaki et al., 2008).

As an extracellular serine protease, tPA participates in the regulation and revision of tissue components located outside the cell, leading to alterations in neuroplasticity (Kozai et al., 2007). One study found that reactive astrocytes express tPA following trauma to the spinal cord, and tPA inhibitors injected into the spinal canal successfully inhibit mechanical pain triggered by dorsal root ligature (Kozai et al., 2007).

### Growth factors

In recent years, BDNF, which binds to TrkB, has been found to be closely associated with astrocyte activation in a rat model of spinal nerve ligation. In certain experimental studies, the BDNF inhibitor ANA-12 was found to inhibit astrocyte activation in the dorsal horn of the spinal cord and subsequently alleviate or even inhibit the onset of mechanical nociceptive hypersensitivity. Increased BDNF levels and proinflammatory cytokines have also been observed following astrocyte activation (Chiang et al., 2012). Similarly, mechanical pain alterations have been reversed using fluorotic acids that inhibit astrocyte activation to downregulate BDNF (Ding et al., 2020). These actions are all accomplished through the binding of BDNF to TrkB.T1, the only TrkB receptor subtype expressed on astrocytes (Wu et al., 2013). BDNF produced by astrocytes has also been found to enhance NMDA receptors in excitatory neurons and decrease inhibitory neuronal activity by accomplishing depolarization displacement of the transient reversal potential (E_GABA_) and decreased conductance of presynaptic gamma-aminobutyric acid (GABA). These provide BDNF the ability to enhance the synaptic activity of excitatory neurons, thus promoting the development of neuropathic pain (Zhou et al., 2021).

## Supraspinal astrocytes in neuropathic pain

As neuroplasticity advances in the spinal cord and ascending routes reach the upper spinal regions, the production and transmission of action potentials are facilitated, resulting in abnormal manifestations of pain behaviors. Of particular interest are the PAG and rostral ventromedial medulla (RVM), which play a key role in nociceptive regulation (Dubový et al., 2018). Belief in the elevated involvement of the PAG and RVM in nociceptive modulation is also growing. Other studies have revealed that unilateral sterile CCI (sCCI) or complete SNT (CSNT) for several weeks induces significant bilateral astrocyte activation in the dlPAG and vlPAG. Astrocyte activation is more extensive in CSNT than in sCCI, and more extensive astrocyte activation is also observed in the RVM. Although the ability of CSNT to trigger astrocyte transformation in the PAG and RVM into an active state is stronger, the ability of the two kinds of sciatic nerve damage to trigger an active state of microglia in upper spinal tectonics is similar. Furthermore, this suggests that reactive astrocytes in the PAG and RVM can be sustained to promote downstream structural groups and consequently maintain neuropathic pain, whereas reactive microglia tend to be involved in response to persistent peripheral nerve injuries. CCL2 secreted by neurons and astrocytes after PAG and RVM damage has the potential to trigger reactive microglia mediated by CCR2 (Dubový et al., 2018). Astrocyte activation in the PAG and RVM contributes indispensably to the progression and sustainment of neuropathic pain.

## Astrocyte–microglia and astrocyte–neuron interactions

CCL2 and CXCL1 have been found to be expressed in spinal cord astrocytes, act on CCR2 and CXCR2 in spinal cord neurons, and thus potentially play a role in increasing excitatory synaptic transmission. Studies on electrophysiological aspects found CCL2 to immediately enhance NMDA-induced currents and increase the frequency of excitatory postsynaptic currents in spinal cord lamina II neurons. Co-stimulation of spinal cord neurons with CCL2 and GABA resulted in a dose-dependent and rapid decrease in GABA-induced inward currents (Zhang et al., 2017). CCL7 (monocyte chemotactic protein 3), produced by spinal astrocytes, has also been shown to act on CCR2 in microglia and induce the activation of spinal microglia (Imai et al., 2013). These findings demonstrate that astrocytes do not act independently. They are inextricably linked to neurons and microglia and concertedly contribute to the development of neuropathic pain. This aspect of research warrants deeper exploration.

## New analgesic drugs targeting astrocytes

To date, no definitive effective treatment options for neuropathic pain are available worldwide. The most frequently used drugs include tricyclic antidepressants, ion channel-modulating drugs (gabapentin, pregabalin), and some anticonvulsants. These drugs only provide pain relief but do not completely eradicate neuropathic pain, and they often produce a number of side effects (O’Connor and Dworkin, 2009; Zychowska et al., 2015). Other analgesics, such as opioids, are even less effective in the clinical treatment of neuropathic pain (O’Connor and Dworkin, 2009). Although the clinical application of analgesic drugs targeting astrocytes has not yet commenced, targeted treatment of neuropathic pain *via* astrocytes may emerge as a new alternative for affected patients. In fact, several experimental studies are already underway. Dexmedetomidine has been found to effectively inhibit HMGB1-mediated astrocyte activation and the TLR4/NF-κB signaling pathway in CCI rats and successfully relieve neuropathic pain in rats (Zhao et al., 2020). Koumine has been found to exert analgesic effects by inhibiting astrocyte activation and the release of proinflammatory cytokines (Jin et al., 2018). Triptolide, the active component of *Tripterygium wilfordii* Hook F, in combination with MK-801, the non-competitive *N*-methyl-D-aspartate receptor antagonist, inhibits astrocyte activation and STAT phosphorylation, consequently exerting synergistic analgesic effects (Wang et al., 2017). All these drugs inhibit the contributory process of astrocytes to neuropathic pain to a certain extent and are expected to be new protocols and strategies for the treatment of neuropathic pain. However, most of these drugs remain at the animal testing stage, and their specific application methods and exact clinical effects require further investigation and exploration.

## Conclusion

To conclude, astrocyte activation and the changes in the signaling pathways and molecular mechanisms involved in the spinal cord and supraspinal structures contribute indispensably to the progression and sustainment of neuropathic pain. Elucidating the mechanism underlying astrocyte activation is of great significance to the progress and development of pain management worldwide. Currently, the exploration of new targeted pain treatments in the wake of opioid overdependence and overuse is of particular importance. Research on the role played by astrocytes in neuropathic pain is expected to realize the clinical transformation of animal experimental research, which is of great significance for human happiness and quality of life.

## Author contributions

XM: conceptualization, writing-reviewing, and editing. TC: writing-original draft preparation. ZX: supervision. All authors have approved the final version of the manuscript to be submitted.
